# Supplementary material for: The circadian oscillator analysed at the single‐transcript level
Source: Mol Syst Biol. 2021 Mar 15;17(3):e10135. doi: 10.15252/msb.202010135 (PMC7957410; doi:10.15252/msb.202010135)
Supplement: Supplementary file 1 — Appendix [file MSB-17-e10135-s003.pdf]

The circadian oscillator observed at the single-transcript level - Appendix

Nicholas E. Phillips*, Alice Hugues*, Jake Yeung, Eric Durandau, Damien Nicolas, Felix Naef

Contents

1 Model parameters for the simplified stochastic model **2**

2 Parameter estimates **3**

2.1 Model M1 3

2.2 Model M2 3

2.3 Model M3 4

2.4 Model M4 4

3 Appendix Figures **5**

1 Model parameters for the simplified stochastic model

Here we show the parameters for the simplified stochastic model of the *Nr1d1*/*Cry1*/*Bmal1* reaction network that is used in Appendix Figure S13.

Parameter	Description	Units	Value
α_p	Translation rate	hours^{-1}	10
δ	mRNA and protein degradation rate	hours^{-1}	log(2)/2
P_0	Protein threshold in Hill function		1500
n	Hill coefficient		2.5
$k_{\mathrm{off,Cry1}}$	Gene deactivation rate *Cry1*	hours^{-1}	9.6
$\alpha_{\mathrm{M,Cry1}}$	Basal transcription rate *Cry1*	hours^{-1}	190
$k_{\mathrm{off,Nr1d1}}$	Gene deactivation rate *Nr1d1*	hours^{-1}	4.9
$\alpha_{\mathrm{M,Nr1d1}}$	Basal transcription rate *Nr1d1*	hours^{-1}	690
$k_{\mathrm{off,Bmal1}}$	Gene deactivation rate *Bmal1*	hours^{-1}	11
$\alpha_{\mathrm{M,Bmal1}}$	Basal transcription rate *Bmal1*	hours^{-1}	160

Appendix Table S1: Parameters of the simplified stochastic model and their values.

2 Parameter estimates

Here we show the posterior parameter estimates for each model. The values quoted correspond the median (i.e. 50% percentile), and the 5% and 95% percentiles are shown in the brackets.

2.1 Model M1

Parameter	Description	Units	*Nr1d1*	*Cry1*	*Bmal1*
Burst size (b_g)	Average #mRNA produced during each burst	#mRNA	14.6 (13.9,15.4)	3.92 (3.77,4.09)	9.09 (8.69,9.55)
Frequency scale (γ_g)	Rescales the function $m_g(t)$ to burst frequency	mRNA half-life^{-1}	0.0682 (0.0652,0.0714)	0.254 (0.244,0.264)	0.109 (0.104,0.114)

Appendix Table S2: The 50% percentile (5%, 95%) for posterior parameter samples for model M1

2.2 Model M2

Parameter	Description	Units	*Nr1d1*	*Cry1*	*Bmal1*
Burst size (b_g)	Average# mRNA produced during each burst	#mRNA	14 (13.3,14.8)	3.32 (3.18,3.47)	7.04 (6.71,7.41)
Frequency scale (γ_g)	Rescales the function $m_g(t)$ to burst frequency	mRNA half-life^{-1}	0.0723 (0.0691,0.0757)	0.306 (0.293,0.319)	0.142 (0.136,0.149)
Area exponent (β_g)	Scaling of burst size with cell area		0.513 (0.448,0.578)	0.64 (0.606,0.676)	0.733 (0.692,0.773)

Appendix Table S3: The 50% percentile (5%, 95%) for posterior parameter samples for model M2

2.3 Model M3

Parameter	Description	Units	Posterior
λ_g	Scaling of oscillation amplitude		1.05 (1.01,1.12)

Appendix Table S4: The 50% percentile (5%, 95%) for posterior parameter samples for model M3

2.4 Model M4

Parameter	Description	Units	*Nr1d1*	*Cry1*	*Bmal1*
Mean burst size (μ_g)	Mean #mRNA produced during each burst (log space)	log(#mRNA)	2.58 (2.56,2.61)	1.18 (1.16,1.19)	1.94 (1.93,1.96)
Std dev of burst size (σ_g)	Standard deviation of burst size (log space)	log(#mRNA)	0.346 (0.308,0.383)	0.221 (0.198,0.244)	0.135 (0.102,0.165)

Parameter	Description	Units	*Bmal1/Cry1*	*Nr1d1/Cry1*
ρ	Correlation in cell-specific burst size		0.861 (0.719,0.947)	-0.916 (-0.968,-0.832)

Appendix Table S5: The 50% percentile (5%, 95%) for posterior parameter samples for model M4

3 Appendix Figures

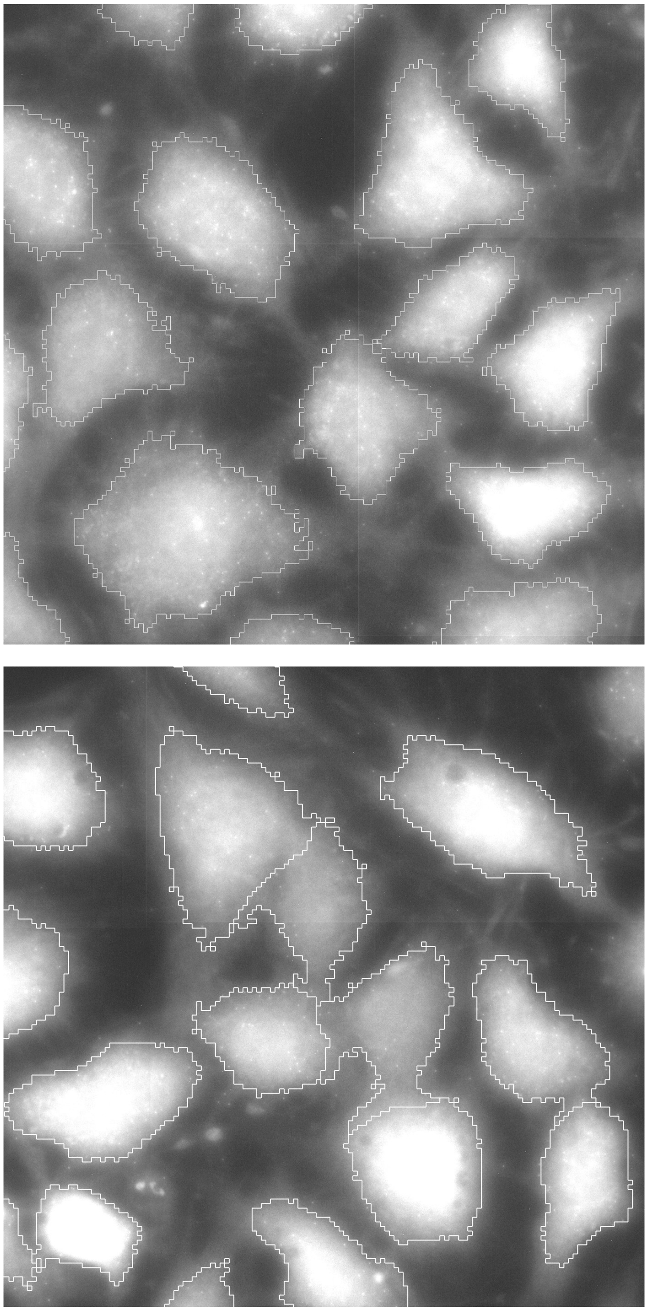

Appendix Figure S1: Examples of the cell segmentation. Cell boundaries, shown in grey, are displayed on the sum Z-projection of the red channel used for the segmentation.

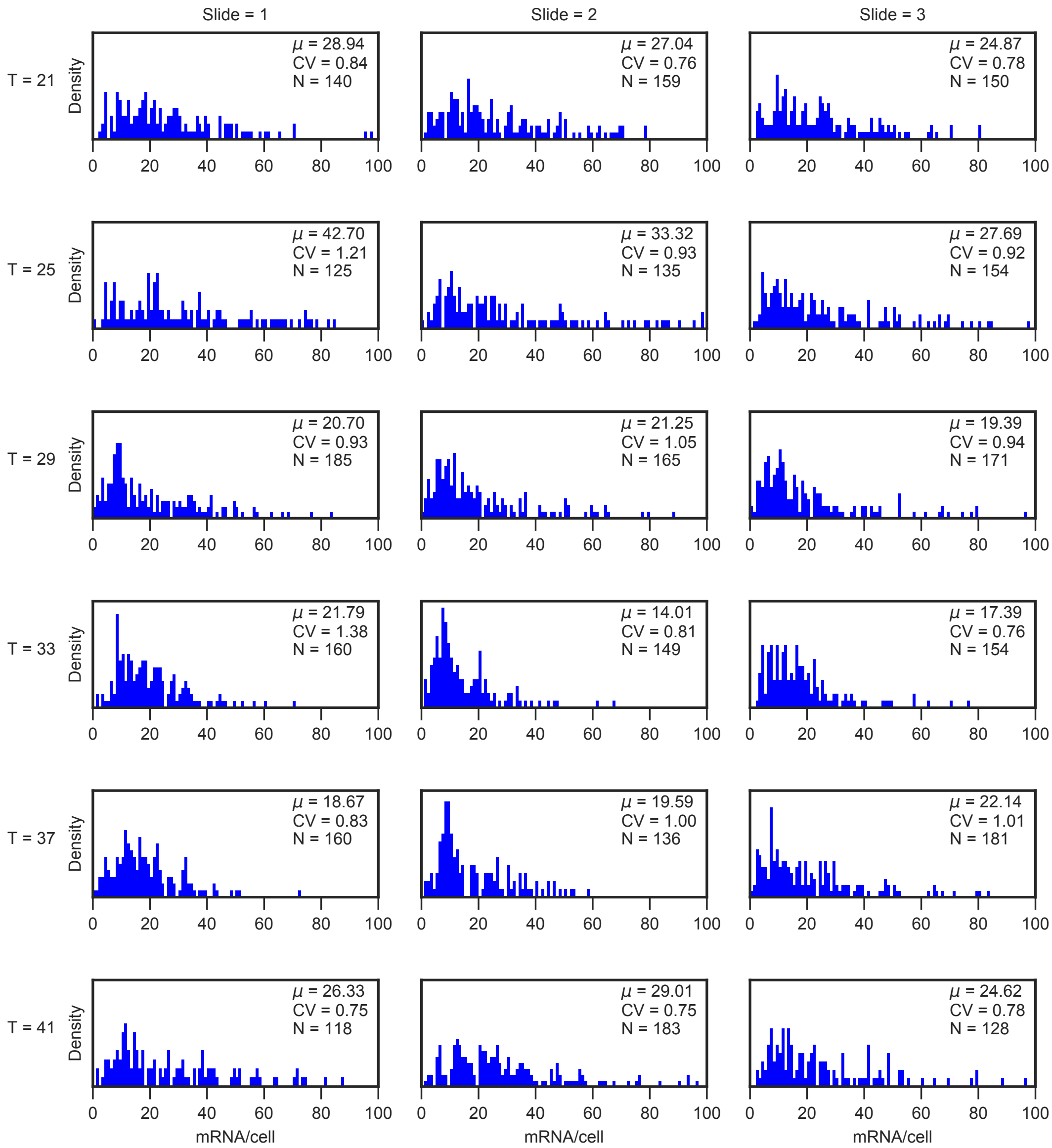

Appendix Figure S2: Distributions of *Nr1d1* transcripts for all time points and all slide replicates (Methods) measured for the *Nr1d1*/*Cry1* smFISH pair. Time T represents hours after synchronisation with Dex. μ represents the mean of the distribution, CV represent the coefficient of variation (standard deviation / mean), and N is the number of cells.

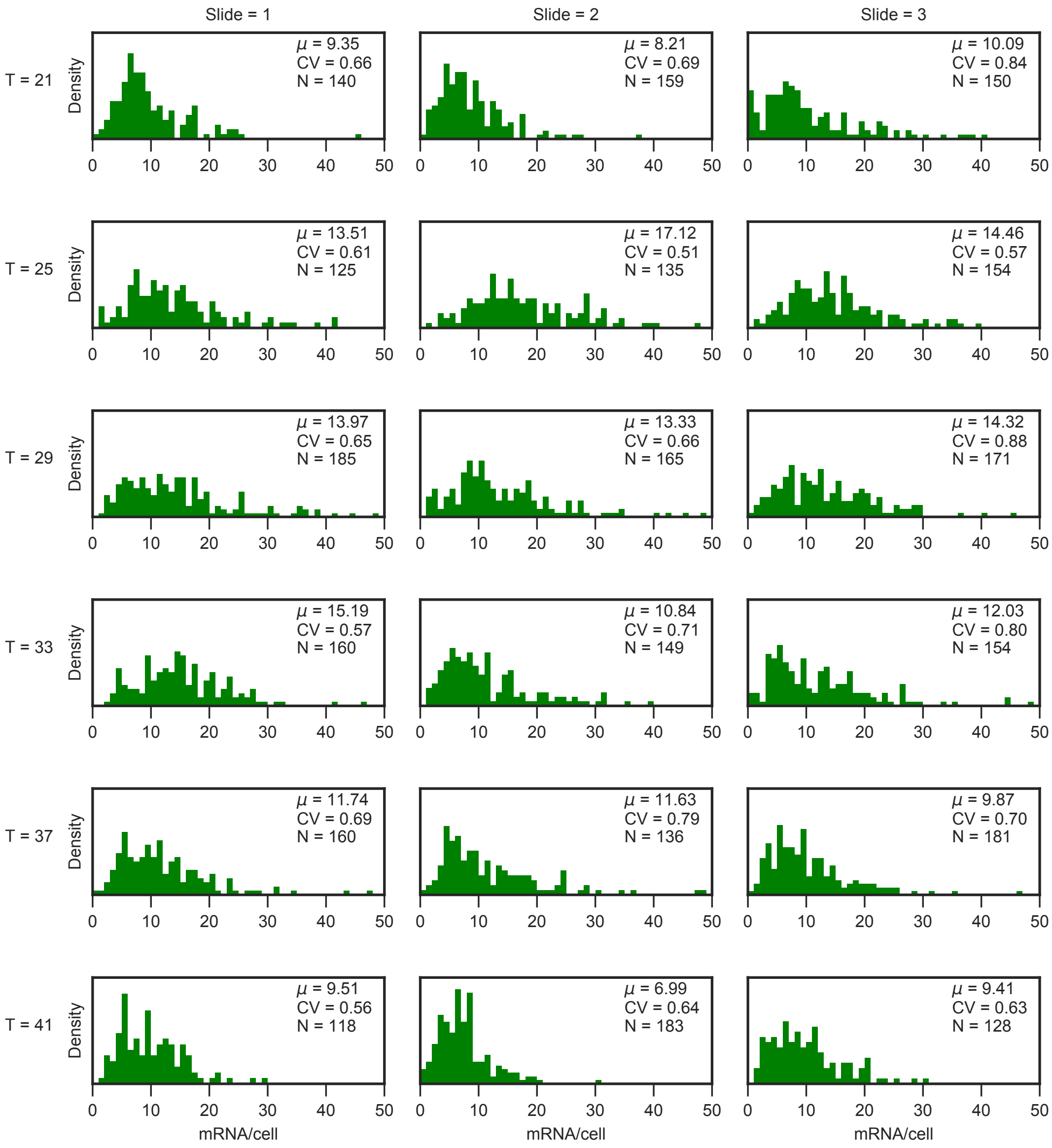

Appendix Figure S3: Distributions of *Cry1* transcripts for all time points and all slide replicates (Methods) measured for the *Nr1d1*/*Cry1* smFISH pair. Time T represents hours after synchronisation with Dex. μ represents the mean of the distribution, CV represent the coefficient of variation (standard deviation / mean), and N is the number of cells.

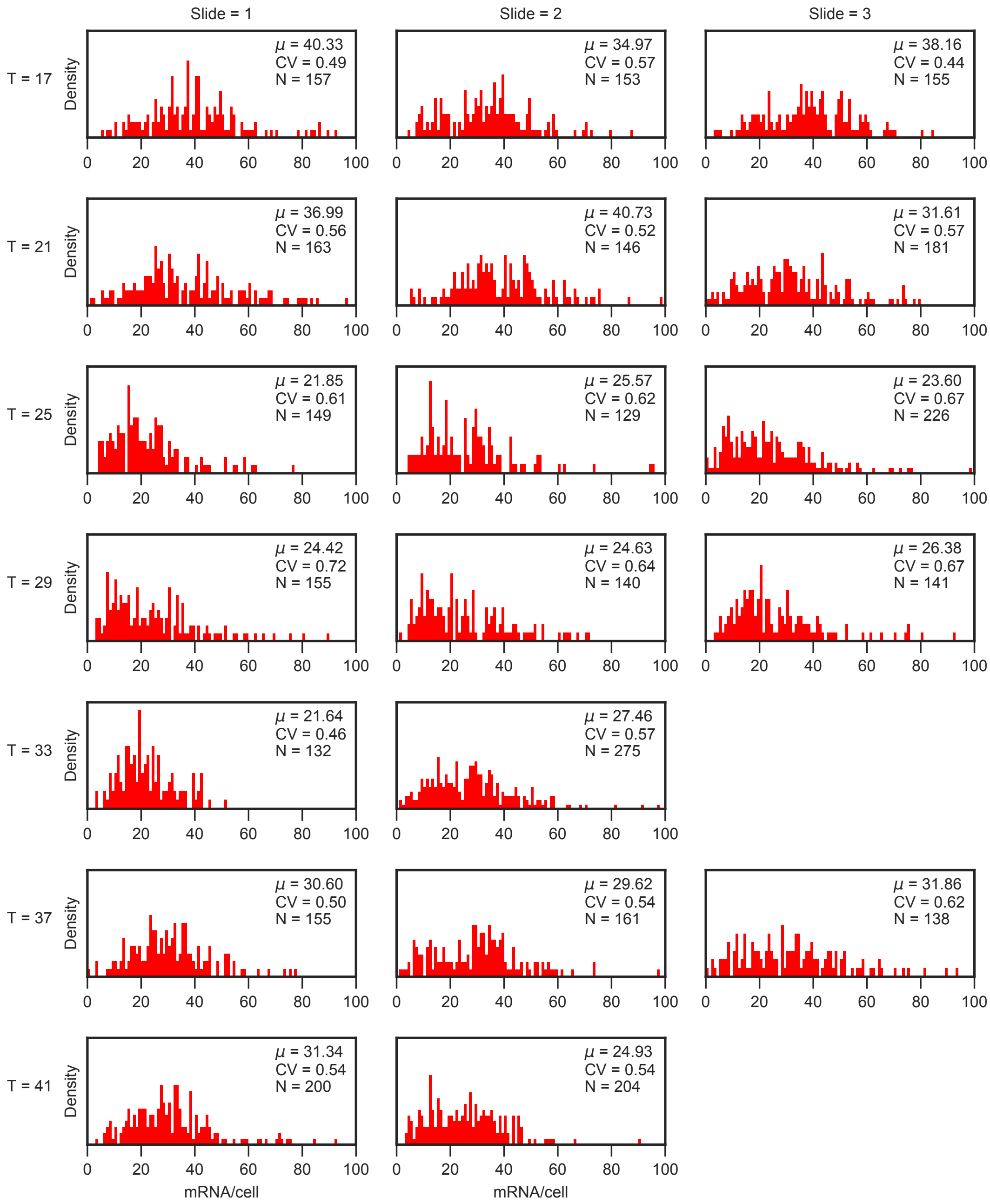

Appendix Figure S4: Distributions of *Bmal1* transcripts for all time points and all slide replicates (Methods) measured for the *Bmal1*/*Cry1* smFISH pair. Time T represents hours after synchronisation with Dex. μ represents the mean of the distribution, CV represent the coefficient of variation (standard deviation / mean), and N is the number of cells.

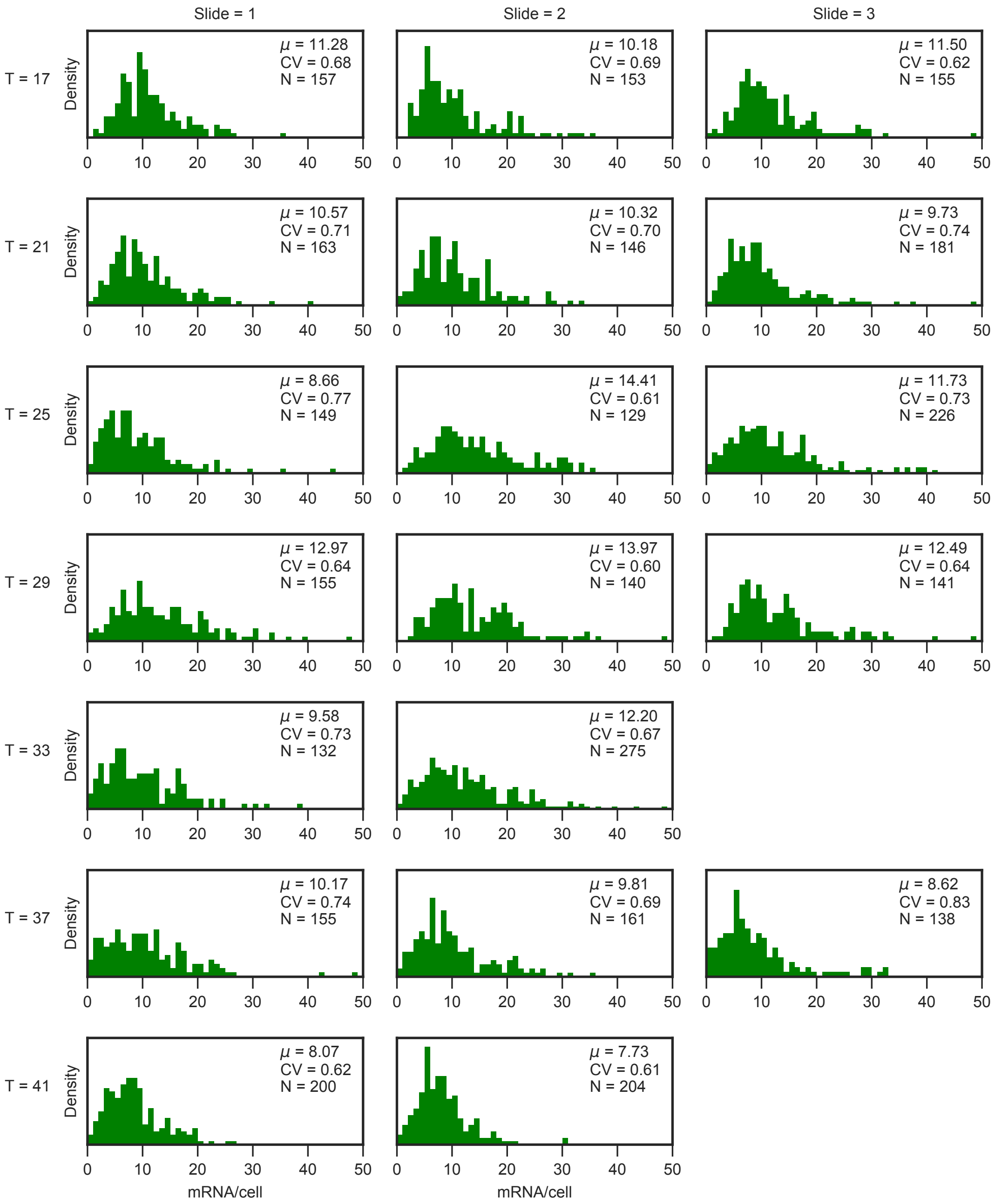

Appendix Figure S5: Distributions of *Cry1* transcripts for all time points and all slide replicates (Methods) measured for the *Bmal1*/*Cry1* smFISH pair. Time T represents hours after synchronisation with Dex. μ represents the mean of the distribution, CV represent the coefficient of variation (standard deviation / mean), and N is the number of cells.

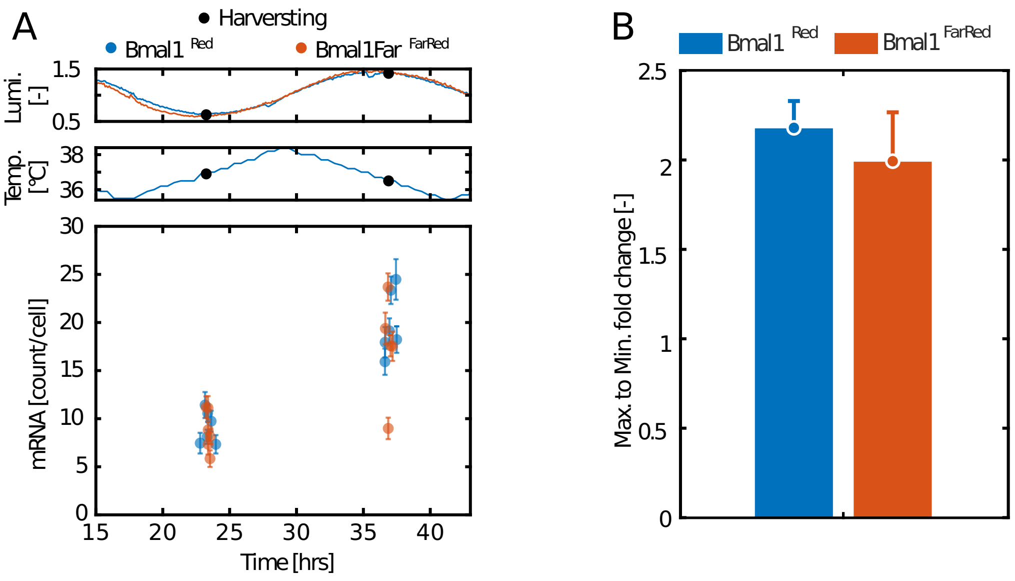

Appendix Figure S6: Amplitude of oscillations with temperature cycle synchronisation. Using a stable Bmal1-sLuc2 NIH3T3 cell line (clone M, Nicolas, 2018), the circadian clock was synchronised using temperature cycles and then split and seeded on a 3.5 cm a Petri dish before recording with an Actimetrics LumiCycle. Note the recording is performed under constant (37°C) temperature. The raw luminescence signal was corrected as described in (Saini et al., 2012). smFISH was performed using probes targeting two non-overlapping regions of *Bmal1* transcripts ($\text{Bmal1}^{\text{Red}}$, $\text{Bmal1}^{\text{FarRed}}$) on three independent cultures of WT NIH-3T3 cells. (A, top) Corrected luminescence from NIH-3T3 cells expressing Bmal1-luciferase. (A, middle) Temperature profile recorded by the Memert incubator. (A, bottom) Mean mRNA count for each replicate. Error bars represent the standard error. (B) Peak-to-trough fold change. Error bars are standard deviation.

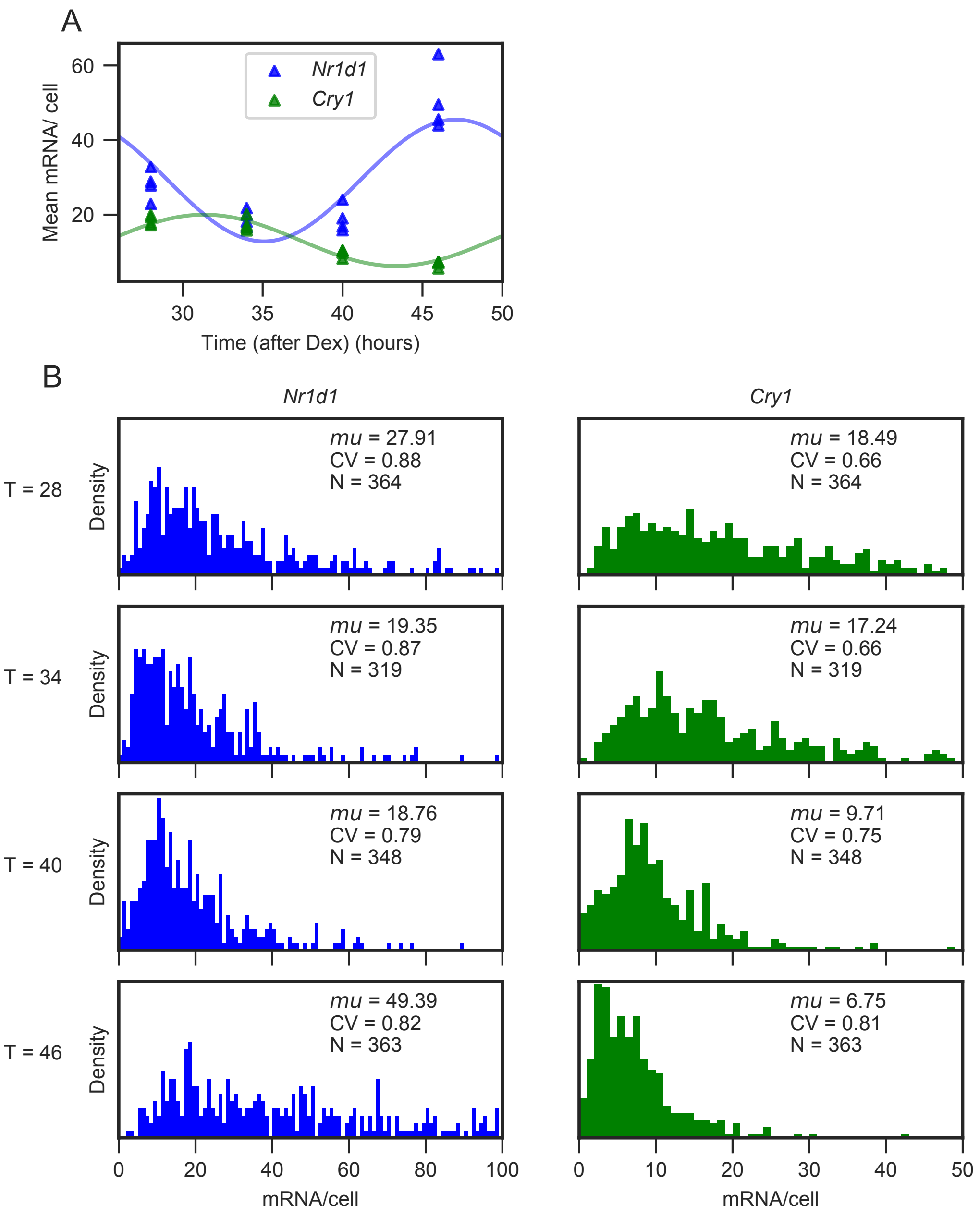

Appendix Figure S7: An independent experiment for the *Nr1d1*/*Cry1* pair shows that the oscillatory mRNA patterns are reproducible. A) Triangles show the mean mRNA number in four images from 2 independent slides. Lines show the least squares regression fit using a cos function. B) Distributions of *Nr1d1* and *Cry1* transcripts at 28, 34, 40 and 46 h after synchronisation with Dex. μ represents the mean of the distribution, CV represent the coefficient of variation (standard deviation / mean), and N is the number of cells at the given time point.

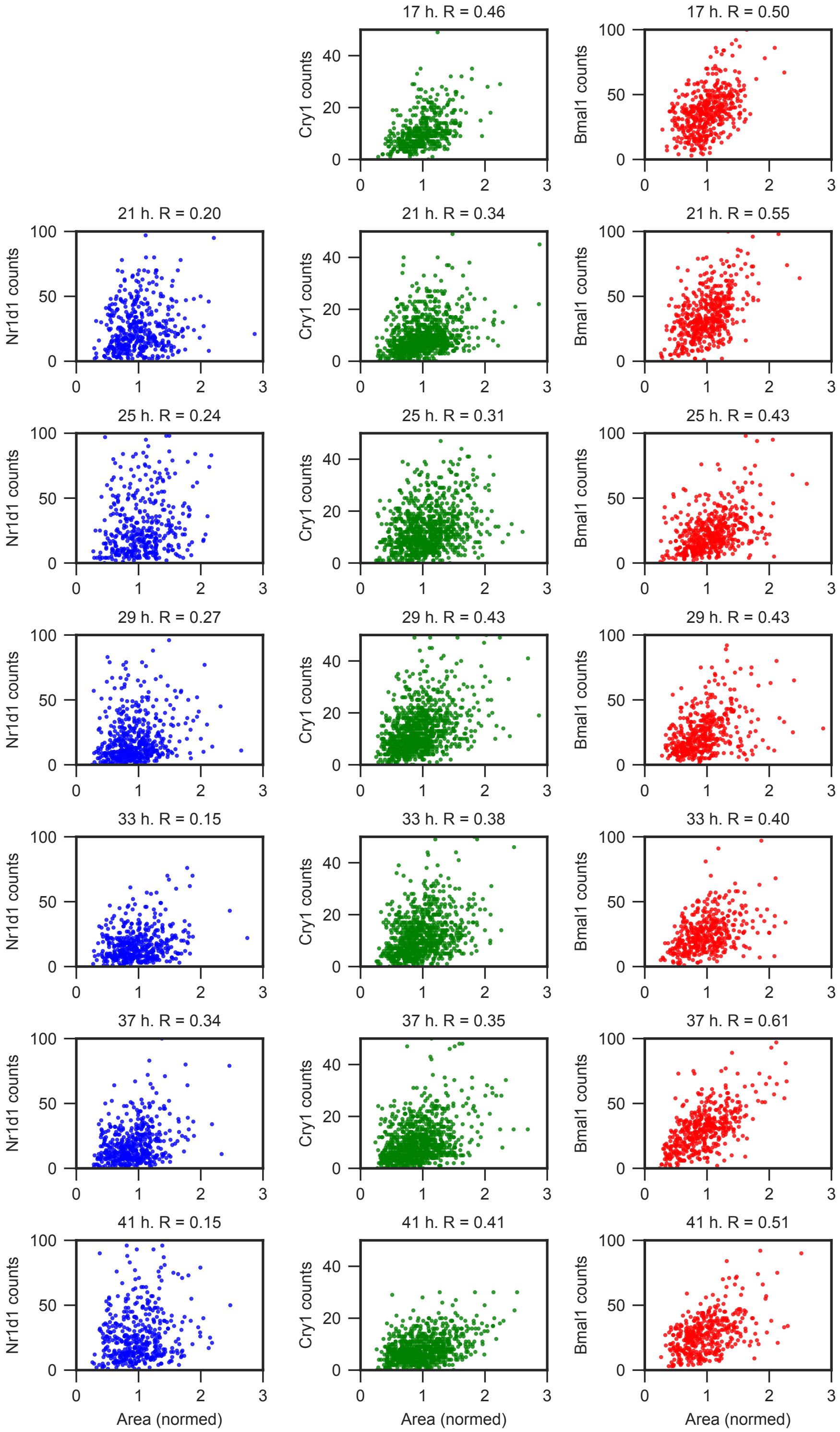

Appendix Figure S8: mRNA count number as a function of area for *Nr1d1*, *Cry1* and *Bmal1* at each time point. The area is normalised such that the mean is 1. R is the Pearson correlation coefficient.

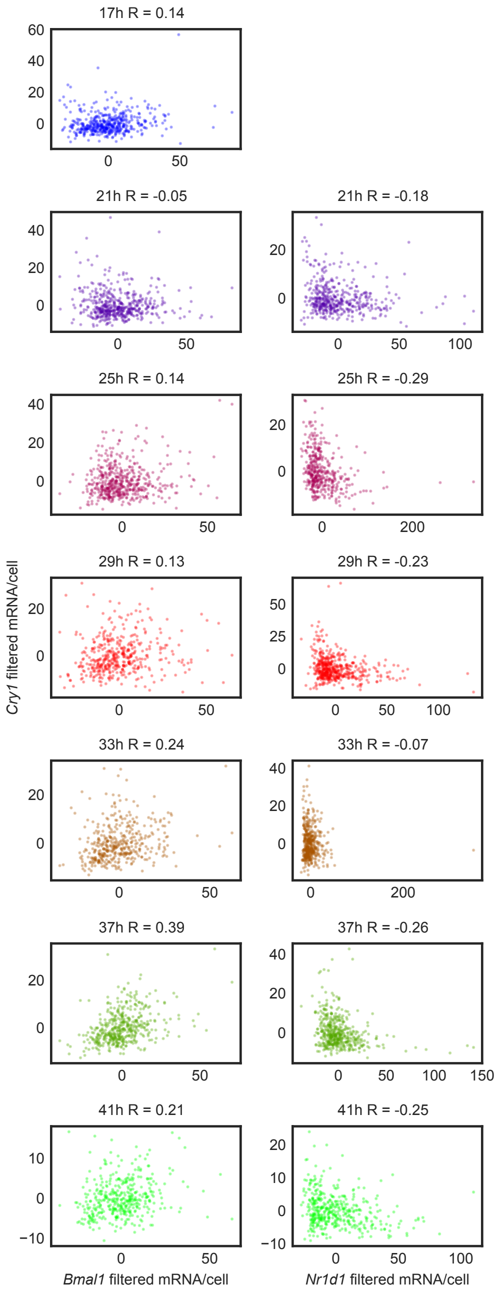

Appendix Figure S9: The correlation between genes after regressing out the effect of cell area. At each time point the linear regression $y = \alpha(\text{Area})^{\beta}$ is fitted to each gene. This function y is then subtracted from the raw mRNA counts to give the filtered values. Each dot corresponds to a single cell, the time represents the number of hours after Dex synchronisation, and R represents the Pearson correlation coefficient.

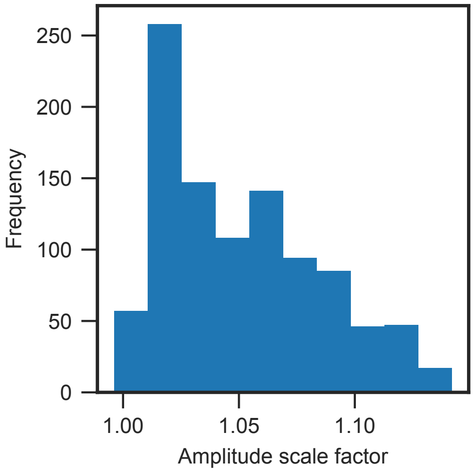

Appendix Figure S10: Posterior parameter estimates for the amplitude scale factor λ in model M3 (described in Methods). A scale factor of 1.05 corresponds to a 5% increase in amplitude relative to model M1.

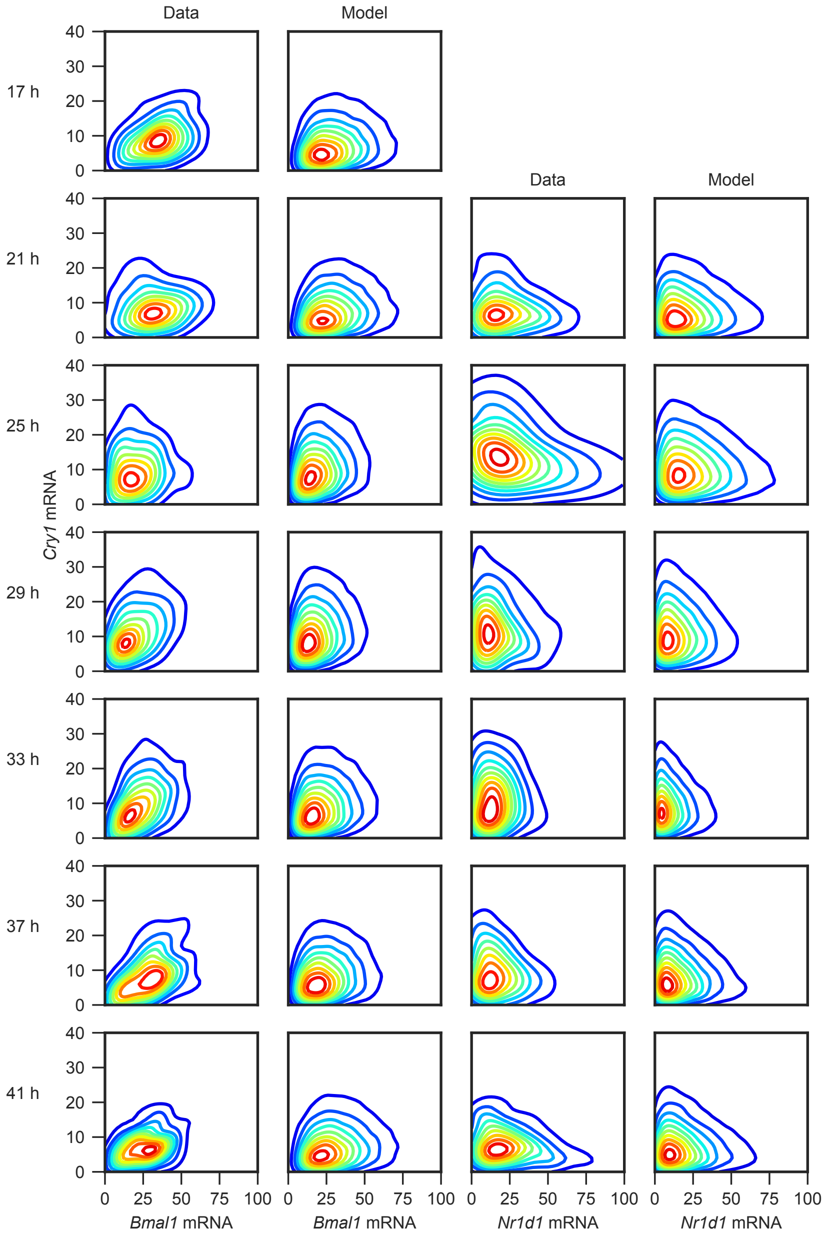

Appendix Figure S11: Comparison of the kernel density estimates of the data with model M4 for all time points. To estimate the probability from the model, the model was simulated 15 times using the posterior mean parameter values combined with the measured cell areas. Note that the same data is also shown in Figure 2C.

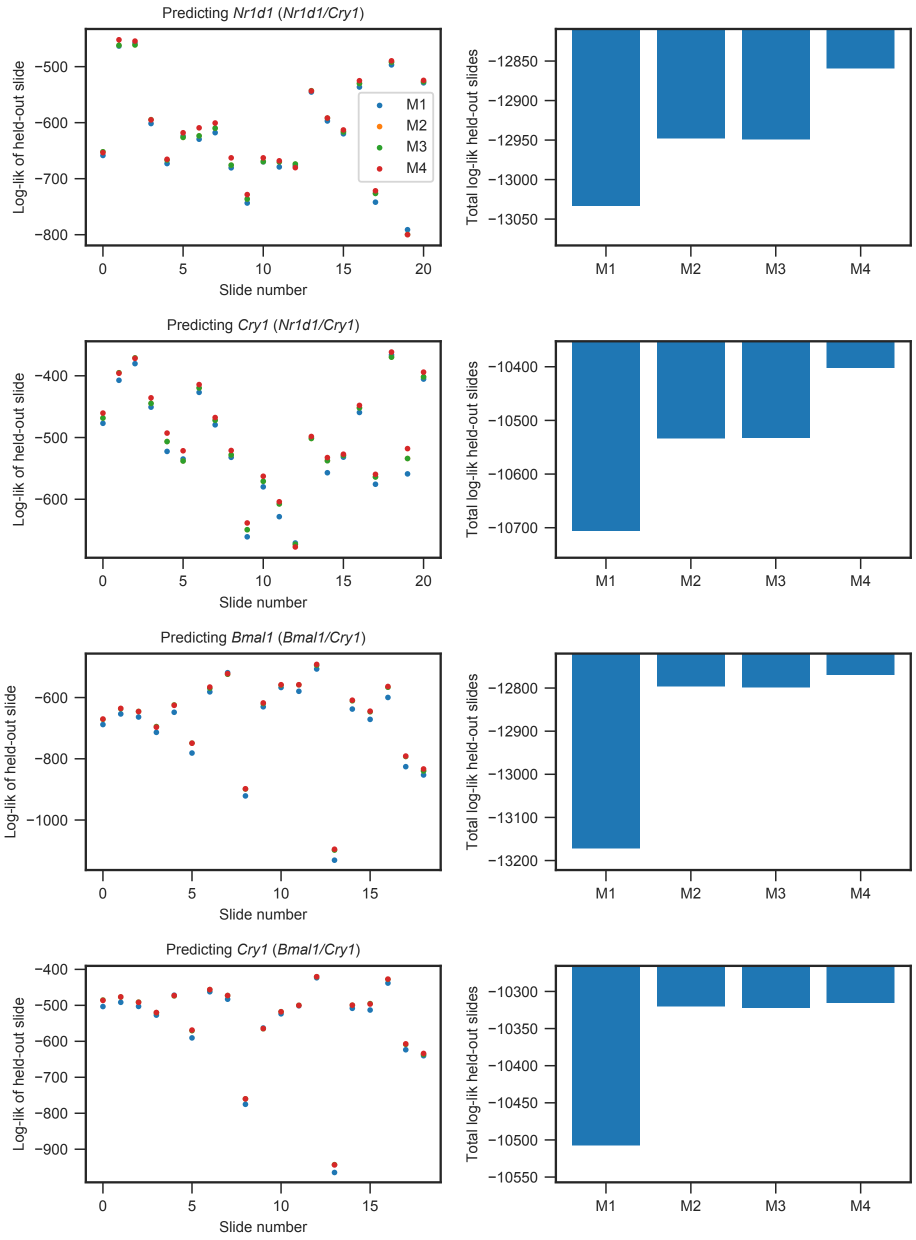

Appendix Figure S12: Leave-replicate-out cross validation. A training set was created by removing the data from one gene for one test replicate. After training each model on the data, the likelihood of observing the test data was calculated by using the mean posterior parameter estimates from the training data. Left column shows the log-likelihood of the test data when each slide is used as the test slide for each model M1-M4. Right column shows the sum of the log-likelihood over all test slides.

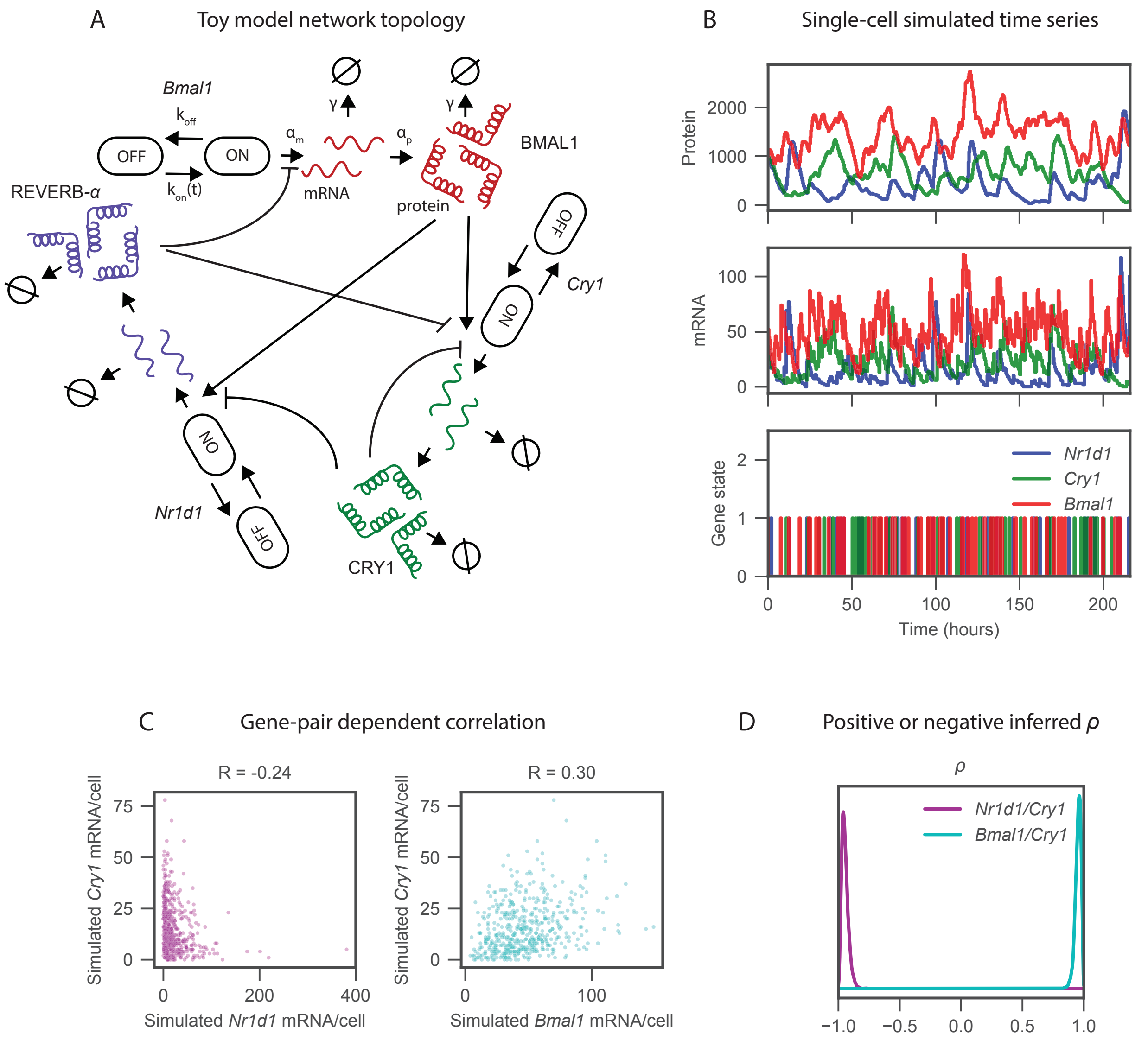

Appendix Figure S13: A simple dynamic model of the circadian clock. A) The network topology used in the model. The transcription rate of each gene is a function of the protein levels in the network, and an oscillatory function $k_{\text{on}}(t)$ controls the gene activation rate. See Methods for a full description of the model. B) An example single-cell time series generated by the model. The initial condition is $X_0 = [0, 1, 30, 1000, 0, 0, 1, 30, 1000, 0, 0, 1, 30, 1000, 0]$. C) The correlation of mRNA between different genes at time T=41h for 500 simulated cells. D) The posterior distribution of the parameter ρ using the simulated mRNA profiles (500 simulated cells) at T = 17, 21, 25, 29, 33, 37, 41h.

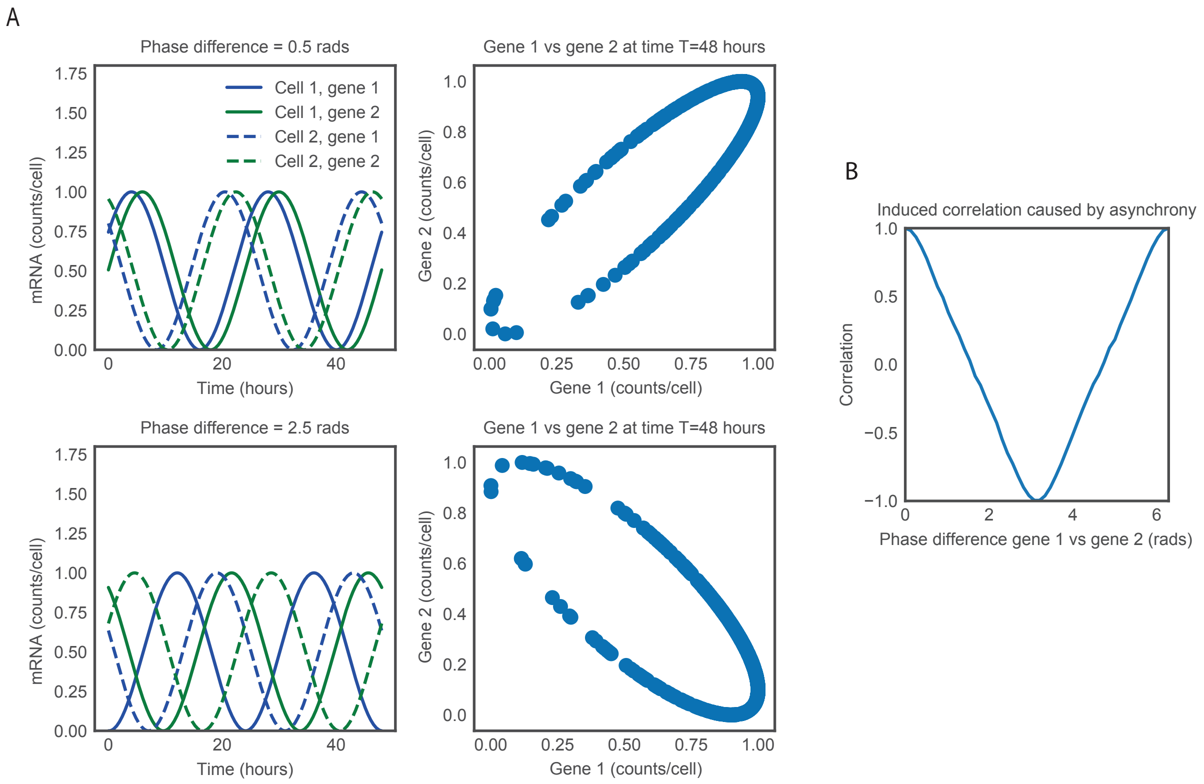

Appendix Figure S14: Illustration of the effects of asynchrony on the mRNA correlation between two different genes. A, top left) When the two genes have a small phase difference and when the phase of cells is spread due to asynchrony, the expression of the two genes is co-ordinated i.e. genes have peak and trough expression simultaneously. A, top right) A snapshot at a single timepoint shows that mRNA levels are positively correlated between the two genes. A, bottom left) When the two genes have a large phase difference (i.e. close to anti-phase), the expression of the two genes are opposite. A, bottom right) A snapshot at a single timepoint shows that mRNA levels are negatively correlated between the two genes. B) The induced correlation caused by imperfect synchronisation as a function of the phase difference between the two genes.
